# Timing matters: Rhizobia strain rankings based on host biomass shift between early and late harvests

**DOI:** 10.17912/micropub.biology.002179

**Published:** 2026-06-29

**Authors:** Alyssa Briggs, Renee Petipas, Xiufen Li, Maren L Friesen, Chandra N Jack

**Affiliations:** 1 Biology, Clark University, Worcester, MA, US; 2 Plant Biology, University of Vermont, Burlington, VT, US; 3 Plant and Environmental Sciences, New Mexico State University, Las Cruces, NM, US; 4 Plant Pathology, Washington State University, Pullman, WA, US

## Abstract

In the legume-rhizobia mutualism, symbiotic success changes with time, complicating early strain quality evaluations. We measured host biomass at three and six months after inoculation of bearded clover ( *Trifolium barbigerum* ) with 77 strains of *Rhizobium leguminosarum* . Across timepoints, strain performance rankings based on host biomass varied: some top strains at three months later declined, whereas initially low-ranking strains ultimately surpassed them. This suggests biological tradeoffs in the timing of nitrogen fixation and the allocation of the host resources. Our results highlight that symbiont function can vary over time and that single timepoint data collection risks inaccurately identifying long-term beneficial strains.

**Figure 1. Rank and biomass distributions between 3- and 6-month harvests:** A) Colored diamond symbols and dashed lines indicate the Top 10, Bottom 10, and Other groups among the 77 strains and show their mean ranks at each harvest. Each line traces a strain’s change in rank over time, while the colored lines and diamond symbols highlight how the three groups differ in their average trajectories. Several Bottom 10 strains at three months improved their relative performance by six months, whereas some Top 10 strains declined in rank over the same period. B) Biomass of plants inoculated by strains from each group at each harvest shows variation between groups.

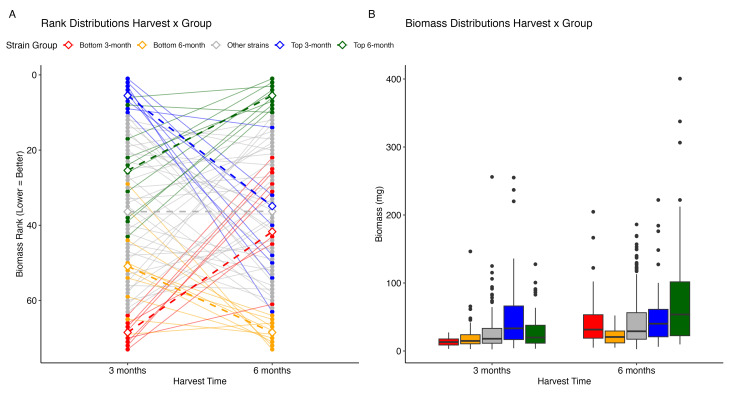



## Description

The success of the legume–rhizobia mutualism depends on reciprocal exchange of carbon and nitrogen, with legumes relying on rhizobia to reduce atmospheric nitrogen to ammonia that fuels host growth and reproduction (Lindström & Mousavi, 2020; Simms & Taylor, 2002). However, the benefits provided by particular rhizobia may not be constant over time (Heath & Tiffin, 2009). A strain that promotes early host growth may not sustain benefits later in development, while a slower-starting strain may yield greater long-term gains (Denison & Kiers, 2004). This raises the question of how harvest timing affects evaluations of symbiotic effectiveness and whether rhizobia performance in promoting host biomass changes over time.

We monitored shoot biomass of *Trifolium barbigerum* singly inoculated with one of 77 *Rhizobium leguminosarum* strains at two time points, three and six months after planting. These time points were chosen to capture early vegetative growth and later reproductive investment. We asked whether partner quality, quantified as host biomass, remains stable or instead shifts through temporal changes in rhizobia function and host response.

We ranked strains by mean host biomass at each harvest and grouped them into three performance categories (Top 10, Bottom 10, and Other; Figure 1A ). We found that the biomass of the plants within each group and harvest were significantly different (Group: F _4,1485_ = 34.183, p < 0.001; Harvest: F _1, 1485_ = 83.45, p < 0.001; Group x Harvest: F _4, 1485_ = 18.63, p < 0.001). When examining by rank, many strains shifted markedly between three and six months. Some Bottom 10 strains at three months rose into higher-ranked positions, whereas several early Top 10 strains dropped out of that group by six months. To quantify how strongly rankings changed over time, we compared strain ranks between harvests. Across all 77 strains, ranks at three and six months showed only a weak, nonsignificant association (Spearman ρ = 0.20, p = 0.084), indicating that early rank was a poor overall predictor of later rank. Within each subset of strains that were in the Top 10 or Bottom 10 at either harvest, correlations between early and late ranks were also nonsignificant (Top at 3 months: ρ = −0.24, p = 0.51; Bottom at 3 months: ρ = 0.10, p = 0.78; Top at 6 months: ρ = 0.33, p = 0.35; Bottom at 6 months: ρ = −0.43, p = 0.21), reinforcing that extreme early performance did not reliably predict later relative rank. Of the 10 strains in the Top 10 at three months, only 2 remained in the Top 10 at six months and 8 shifted into the intermediate “Other” category, while none fell into the Bottom 10. Likewise, only 2 of the Bottom 10 strains at three months remained in the Bottom 10 at six months, with 8 moving into the Other category. Looking from the six-month harvest backwards, 8 of the 10 top strains and 8 of the 10 bottom strains were classified as “Other” at three months. These results show that single early time point measurements are a poor guide to which rhizobia will ultimately rank among the most beneficial for host biomass.

These patterns indicate that strain performance is best viewed as a trajectory over time rather than a fixed property revealed by a single harvest. These findings suggest that the consequences of strain choice for host performance emerge over the course of the interaction, and that early screens alone are likely to miss many strains that ultimately become highly beneficial or particularly poor partners.

Nitrogen is important to *T. barbigerum* at all stages, but its fitness consequences differ: early nitrogen promotes vegetative growth and competitive establishment, whereas later nitrogen contributes more directly to reproductive output (Marrou et al., 2018). Nitrogen fixation is metabolically expensive, so traits that make strains highly effective fixers can come at a cost to other components of fitness, such as growth, competitiveness, or persistence outside nodules (Burghardt, 2020; Burghardt & diCenzo, 2023). Some strains may be favored to secure nodule space and host carbon early in the season even if they provide relatively modest long-term benefits, whereas others may incur higher costs to maintain fixation over longer periods, better matching the host’s reproductive demands. These tradeoffs are consistent with the shifts in strain rankings we observe.

Because *T. barbigerum* forms indeterminate nodules that remain active and continue growing, with persistent meristems and spatial separation of younger and older nodule tissues, host plants can adjust investment in individual nodules over time (Guinel, 2009; Mendoza-Suárez et al., 2021; Schwember et al., 2019). In these systems, hosts can maintain investment in nodules housing higher-performing rhizobia while reducing support to underperforming nodules, creating the potential for changes in the relative value of different strains as plants progress through their life cycle. As a result, mutualism quality becomes a moving target shaped by host development and rhizobia tradeoffs, making it crucial to evaluate plant-rhizobia interactions at multiple time points rather than relying only on early data collection. For stakeholders using bearded clover as a cover crop or forage, this suggests that choosing rhizobia strains based only on early growth could underestimate the benefits of strains that deliver more nitrogen and biomass later in the season. More broadly, our results suggest that evolutionary tradeoffs in microbial traits, rather than a simple lack of more cooperative strains, constrain durable crop benefits from microbial partners (Denison, 2019). Incorporating time as a core dimension of partner quality may therefore help explain why many mutualisms harbor persistent variation in symbiont benefit, even under strong selection for more cooperative partners.

## Methods

*T. barbigerum* (Accession AL8138) seeds were scarified, surface-sterilized in 6% sodium hypochlorite, germinated on water agar plates, and planted in 153 mL D-pots (Stuewe & Sons, Tangent OR) filled with sterile vermiculite moistened with 50 mL of 1x Fahraeus solution to grow in a Washington State University greenhouse (46.73°N, 117.16°W; Conditions: 16 hour days with temperature range of 18°C - 24°C) from 17-December, 2018 until final harvest 6-June, 2019. Plants were randomly assigned to rhizobia, N-free, or N-supplemented treatments (14 reps for each strain, 60 reps for each control). Three days after planting, and again after three weeks to ensure adequate inoculation, the seedlings were inoculated with either rhizobia or sterile 1/2x PBS and covered with sterile sand to reduce the probability of cross-contamination. Each plant label was also marked with a sticker when inoculated to indicate status and prevent accidental over-inoculation. Two and six weeks after the second round of inoculation, the N-supplemented control plants were inoculated with 1mL of 90 mg/L NH _4_ NO _3_ and all other plants received 1mL of 1/2x PBS. All plants were fertigated with 2 minutes of nutrient water at 0, 3, and 6 weeks post second inoculation.

Seventy-seven strains of *Rhizobium leguminosarum* , previously isolated from *Trifolium* nodules collected near the University of California Davis Bodega Marine Lab, were grown from frozen stock on yeast mannitol agar plates (0.1% yeast extract, 1% mannitol, 0.05% K2HPO4, 0.02% MgSO4, 0.01% NaCl2, 1% CaCO3, 1.5% Bacto-Agar) at 30°C for 3 days. Plates of each strain were rinsed with sterile 1/2x PBS and adjusted to a final concentration of 5*10^7 CFU/mL based on OD. Inoculated plants received 500 microliters of the inoculum.

Plant harvests were conducted at approximately 3 and 6 months after planting. At three months, none of the plants had flowered while 69% had flowered at six months. At each harvest, shoots were separated from roots, roots were washed free of vermiculite, and shoots were dried at 60°C and weighed to obtain biomass. At the three-month harvest (18-22 March 2019), all nodules were additionally removed, counted, dried and weighed. None of the N-free or N-supplemented plants had nodules, indicating that we did not have cross-contamination.

Statistical Analysis: Statistical analyses were conducted in Rstudio using R version 4.5.2 (R Core Team, 2025), lmerTest (Kuznetsova et al., 2017) and the tidyverse package (Wickham H et al., 2019). Host response was quantified as mean shoot biomass per rhizobia strain at each harvest. To restrict analyses to symbiotically active partners, strains with a mean of less than one nodule at the three-month harvest were excluded.

For each harvest, strains were ranked by mean shoot biomass (rank 1 = highest biomass), and the top and bottom ten strains were identified to define “Top” and “Bottom” groups, with all remaining strains classified as “Other.” These rank-based groups were used to examine temporal consistency and potential tradeoffs in strain performance across harvests. We assessed differences in biomass between groups using a linear mixed model with strain id as a random effect. To quantify temporal stability in performance, Spearman rank correlations were calculated between strain ranks at the first and second harvests for all strains and within each rank-based strain subset (Top at 3 months, Bottom at 3 months, Top at 6 months, Bottom at 6 months).
